# Upregulation of HCFC1 expression promoted hepatocellular carcinoma progression through inhibiting cell cycle arrest and correlated with immune infiltration

**DOI:** 10.7150/jca.84579

**Published:** 2023-05-15

**Authors:** Huaxiang Wang, Meng Yu, Chengkai Yang, Qingsong Li

**Affiliations:** 1 Department of Hepatobiliary and Pancreatic Surgery, Taihe Hospital, Affiliated Hospital of Hubei University of Medicine, Shiyan, Hubei 442000, China.; 2 The Fuzong Clinical Medical College of Fujian Medical University, Fuzhou, Fujian 350025, China.; 3 Department of Critical Care Medicine, Taizhou Central Hospital (Taizhou University Hospital), Taizhou, Zhejiang 318000, China.; 4 Department of Gastroenterology, Taizhou Central Hospital (Taizhou University Hospital), Taizhou, Zhejiang 318000, China.

**Keywords:** Host cell factor 1, Hepatocellular carcinoma, Cell cycle, Proliferation, Immune infiltration.

## Abstract

**Background:** Host cell factor 1 (HCFC1) was reported associated with the progression of a variety of cancers. However, its role in the prognosis and immunological characteristics of hepatocellular carcinoma (HCC) patients has not been revealed.

**Methods:** The expression and prognostic value of HCFC1 in HCC were investigated from the Cancer Genome Atlas (TCGA) dataset and a cohort of 150 HCC patients. The associations between HCFC1 expression with somatic mutational signature, tumor mutational burden (TMB), and microsatellite instability (MSI) were investigated. Next, the correlation of HCFC1 expression with immune cell infiltration was investigated. In vitro, cytological experiments were conducted to verify the role of HCFC1 in HCC.

**Results:** HCFC1 mRNA and protein upregulated in HCC tissues and correlated to poor prognosis. Multivariate regression analysis based on a cohort of 150 HCC patients revealed that high HCFC1 protein expression was an independent risk factor for prognosis. Upregulation of HCFC1 expression was associated with TMB, MSI, and tumor purity. HCFC1 expression showed a significant positive association with B cell memory, T cell CD4 memory, macrophage M0, and a significant positive association with immune checkpoint-related gene expression in the tumor microenvironment. HCFC1 expression negatively correlated to ImmuneScore, EstimateScore, and StromalScore. The single-cell RNA sequencing analysis demonstrated that the malignant cells and immune cells (B cells, T cells, and macrophages) represented high HCFC1 expression in HCC tissues. Functional analysis revealed that HCFC1 was remarkably correlated with cell cycle signaling. HCFC1 knockdown inhibited the proliferation, migration, and invasion capacity while promoting the apoptosis of HCC cells. At the same time, the cell-cycle-related proteins such as Cyclin D1 (CCND1), Cyclin A2 (CCNA2), cyclin-dependent kinase 4 (CDK4), and cyclin-dependent kinase 6 (CDK6) were downregulated.

**Conclusion:** Upregulation of HCFC1 predicted undesirable prognosis of HCC patients and promoted tumor progression through inhibiting cell cycle arrest.

## Introduction

Hepatocellular carcinoma (HCC) is one of the highest morbidity rates of digestive system cancers, and it pressured immense health and economic burden globally, especially in eastern and other low-income countries 1, 2. Viral hepatitis, alcohol, cirrhosis, nonalcoholic fatty liver disease, and metabolic syndrome were known to be prominent risk factors for HCC 3-5. Although the growth of the incidence of HCC has slowed and comprehensive cancer treatment such as immunotherapy and genomically targeted therapies have achieved curative advances, the 5-year mortality rate has not significantly decreased 6, 7. Up to 80% of patients have an advanced stage of HCC during the time of the first visit. Despite substantial efforts have been made to elucidate the molecular mechanism of tumorigenesis and progression of HCC over the past two decades, there is still a lack of clinically effective molecular targets 8-10. The current treatment for early-stage HCC patients was mainly radical surgical resection, supplemented with radiofrequency ablation and transcatheter arterial chemoembolization, which can usually achieve salutatory treatment effects 11. Despite neoadjuvant therapies such as molecular targets and immunotherapies having vigorously developed over the last two decades, the mortality of late-stage patients was not a significant improvement 12. A multitude of previous research had provided promising clues for prognostic biomarkers of HCC, which was mainly linked to the process of the cell cycle but with some limitations for clinical application 13, 14. Therefore, ongoing study to clarify the in-depth molecular mechanisms and identify novel targets was inherently necessary for the treatment.

Host cell factor 1 (HCFC1) plays the role of transcriptional co-regulator in human cells 15. Previous studies reported that up to a quarter of all human promoters was regulated by HCFC1 16. One of the most important functions of HCFC1 was to promote cell proliferation by regulating the cell cycle 17, 18. Abnormal HCFC1 expression was associated with severe neurological defects by disrupting neuronal and neural progenitor cells. Castro VL et al. suggested that mutations in the HCFC1 gene cause syndromic and non-syndromic intellectual disabilities 19. Quintana AM reported that HCFC1 was the key gene to regulating craniofacial development 20. HCFC1 also plays a non-negligible role in various types of cancers, such as renal cell carcinoma, cervical cancer, prostate cancer, and myeloid malignancies 21-23. Dysfunctions of HCFC1-dependent pathways which were regulated by gene regulation by insulin were closely related to tumorigenesis and progression 24. Previous research through high-throughput sequencing found that HCFC1 expression was upregulated in HCC tissues, but its prognostic value was not investigated 25.

This study was proposed to explore the prognostic significance and uncovered the association between HCFC1 expression with immune characteristics. In addition, we aimed to investigate the effects of aberrant HCFC1 expression on biological behavior in patients with HCC with concomitant efforts to elucidate the possible mechanism beneath the same, providing important clues for the potential of HCFC1 as a therapeutic target in HCC. The main shortcoming of this research was the role of HCFC1 expression on HCC validated in vitro assays and the lack of Vivo experimental validations, which needs further experimental investigations.

## Materials and Methods

### HCFC1 mRNA expression data collection

Transcription profiling data downloaded from the Cancer Genome Atlas (TCGA) and Gene Expression Omnibus (GEO) databases (GSE54236 and GSE76427 datasets) were used to investigate the HCFC1 mRNA expression level in HCC and normal liver tissues. The Kaplan Meier plotter database was employed to explore the associations between HCFC1 expression with survival rates 26.

### Patients and HCC samples

We conducted the immunohistochemical (IHC) staining assay on 150 HCC tissue samples from the 900 Hospital of the Joint Logistic Team to analyze the protein level of HCFC1. Corresponding clinicopathological data were collected to investigate the prognostic significance of HCFC1. The HCC samples were obtained from patients who underwent radical resection of HCC between January 2013 and June 2015. The inclusion criteria of this research were: patients over the age of 18, only one tumor lesion or multiple lesions but limited to one hepatic lobe, without extrahepatic metastasis, Child-Pugh class A or B, did not receive radiotherapy or chemotherapy prior to hepatectomy, only underwent a single curative resection, and postoperative pathologic examination verified as HCC. The exclusion criteria were: patients younger than 18 years old, had extrahepatic metastasis, received radiotherapy or chemotherapy before hepatectomy, Postoperative death from non-HCC causes within one week, and postoperative pathologic examination verified as a mixed type of liver cancer. This research was approved by the Human Research Ethics Committee of the 900 Hospital of the Joint Logistics Team and performed in accordance with the principles of the Declaration of Helsinki (Date: February 18th, 2021; Approval number: KY2021PJ218).

### Immunohistochemistry staining

150 formalin-fixed and paraffin-embedded HCC specimens were prepared into 4 nm sections for immunohistochemical assay. The IHC procedure refers to described earlier 27. IHC staining was independently assessed by two experienced pathologists who had no prior knowledge of any information on the patient's clinical condition and diagnosis. The specific antibodies were used as follows: HCFC1 (ab137618; 1:500; Abcam, UK). The sections were subsequently stained by 3,3'-diaminobenzidine (DAB) and hematoxylin. All stainings were assessed based on the five-point scale: 0= no cells stained positive; 1= less than 25% cells stained positive; 2= 26-50%; 3= 51-75%, 4= more than 75% cells stained positive.

### Relationship Between HCFC1 expression with gene mutation and tumor mutational burden (TMB)

Somatic mutation data were downloaded from the TCGA database to perform the tumor mutation analysis. The “oncoplot” R package was employed to identify the differentially mutated genes between HCFC1-high and low expression subgroups. The "maftools" (version 2.8.05) R package was utilized to calculate the tumor mutation burden (TMB), Mutant-allele tumor heterogeneity (MATH), and microsatellite instability (MSI). We next obtained the data on tumor purity, tumor neoantigen, tumor ploidy, homologous recombination deficiency (HRD), and loss of heterozygosity in HCC from the previous article and we then investigated their associations with HCFC1 expression 28.

### Comprehensive Tumor Immune Analysis

The transcription profiling data from the TCGA database were analyzed by the CIBERSORT algorithm to estimate the abundance of each immune cells types. The differential infiltrates abundance between HCFC1 high and low expression subgroups was compared using the Mann-Whitney U test. The "Estimate" R package (version 1.0.13) was employed to calculate the stromal, immune, and ESTIMATE scores of each patient and we then investigated their associations with HCFC1 expression using the "psych" (version 2.1.6) R package. We next compared the expressed differences of immune checkpoint inhibitor-related genes which were most relevant to HCFC1 expression.

### Single-cell RNA sequencing analysis

The single-cell RNA sequencing was analyzed using the Tumor Immune Single-cell Hub 2 (TISCH2) database to explore HCFC1 expressions at the single-cell level including in malignant cells, hepatocytes, stromal cells, and different types of immune cells. TISCH2 provides detailed cell-type annotation at the single-cell level, enabling the exploration of the tumor microenvironment (TME) across different cancer types 29. Three datasets (LIHC_GSE125449_aPDL1aCTLA4, LIHC_GSE1146409, and LIHC_GSE140228_10X) were selected to further investigate the associations between HCFC1 expression with immune infiltration levels 30-32.

### Identification of biological functions and enrichment pathways of HCFC1

We identified the genes that positively correlated with HCFC1 in the LinkedOmics and cBioPortal databases, respectively 33, 34. Overlapping genes with Spearman's correlation value greater than 0.6 in these two databases were identified as co-expressed genes of HCFC1. Next, gene ontology (GO) and the Kyoto Encyclopedia of Genes and Genomes (KEGG) analysis were performed on these co-expressed genes of HCFC1 to identify the potential biological functions and signaling pathways. We performed gene set enrichment analysis (GSEA) using the “clusterprofiler” R package to further investigate the potential biological functions of HCFC1 in HCC. 373 HCC patients from the TCGA database were divided into two subgroups based on the median mRNA expression value of HCFC1. Items of enriched pathways with adjusted p-value<0.05 were selected as significant pathways.

### Reagents and antibodies

The following reagents and antibodies were used in this study: DMEM (GIBCO BRL); fetal bovine serum (FBS, GIBCO BRL, 10099141); Lipofectamine® 3000 reagent (L3000015, Invitrogen, Thermo Fisher Scientific, USA); TRIzol reagent (TaKaRa, 15596018, China); PrimeScript™ RT reagent Kit (TaKaRa, RR037Q, China); CCK-8 kit (Meilune, MA0218, China); DMEM-diluted Matrigel (BD Biosciences, USA); 5% propidium iodide (Med chemExpres, China); Annexin V-PE and PI (Med chemExpres, China); 10% SDS-PAGE (Beyotime, China); PVDF membrane (Bio-Rad Laboratories, USA); 5% skimmed milk (BBI, China); HRP-conjugated Affinipure Goat Anti-Mouse/Rabbit IgG (Proteintech, SA00001-1/SA00001-2, China). The primary antibodies for western blotting were provided in Supplementary Table 1.

### Cell culture and plasmid transfection

Human hepatocyte cell line LO2 (SNL-141, China) and hepatoma cell lines Huh7 (SCSP-526, China) were obtained from the Cell Bank of Chinese Academy of Sciences (Shanghai,). HepG2 (ATCC, HB-8065, USA), and Hep3B (ATCC, HB-8064, United States) were purchased from American Type Culture Collection (ATCC, Manassas, VA, USA). All cell lines were cultured in DMEM supplemented with 10% heat-inactivated fetal bovine serum (FBS) and were maintained in a moist atmosphere at 37 °C with 5% CO2. The shHCFC1 or shRNA-HCFC1 negative control (shCtrl) were transfected into HepG2 and Huh7 cell lines using Lipofectamine® 3000. The sequence for shHCFC1 was the following: 5'-GCTCTATGAGCAAGTGAAT-3'. The sequence for the shCtrl was as follows: 5′-GAUUGGAAAUCAGAGCACUGCC-3′.

### Quantitative polymerase chain reaction (qRT-PCR)

The total RNA from cultured hepatocyte and hepatoma cells was extracted using TRIzol reagent and was reverse-transcribed with the PrimeScript™ RT reagent Kit into cDNA for subsequent PCR assay. The reverse transcription and qRT-PCR methods were accomplished as described earlier 27. Glyceraldehyde-3-phosphate dehydrogenase (GAPDH) was set as the internal control. The 2-ΔΔCt method was employed to determine the relative mRNA level of HCFC1. The sequences of primer pairs were provided in Table 1.

### Cell Counting Kit-8 (CCK-8) assay

CCK-8 assay was employed to detect the cell viability of HepG2 and Huh7 cells. HCC cells were harvested after shHCFC1 transfected 48 hours and then were added to each well of the 96-well microplate. The microplate was placed into an incubator containing 5% CO2 at 37°C for 24, 48,72, and 96 hours. Cell viability was detected using a CCK-8 kit.

### Transwell migration and invasion assay

The migration and invasion capacity of HepG2 and Huh7 cells were assayed using 8.0‑µm pore size Transwell membranes. For migration assay, HCC cells transfected with shHCFC1 or shCtrl were resuspended in a serum-free medium and then planted in the upper chamber of 24-well transwell chambers, and the complete medium was added to the lower chamber. For invasion assay, the chamber was precoated with DMEM-diluted Matrigel. After the cells migrated or invaded for 24 hours, 95 % methanol and 0.1% crystal violet were used to fix and stain the cells, respectively. Finally, a random field of view at ×100 magnification was selected under the microscope to observe and count the number of cells.

### Wound healing assay

A wound-healing assay was employed to further detect the migration capacity of HCC cells. The transfected cells were seeded in 6-well plates and cultured overnight. The cell layer was wounded by a 200μL sterile tip. The cell layer was washed twice with PBS and then the well plate was cultured in a medium without FBS at 37 °C for 48 h. The wound healing was observed and photographed at 0 h and 48 h.

### Cell cycle assay

Transfected cells (1×10^6^ /well) were trypsinized and isolated and then fixed with 75% ethanol at 4°C overnight. Next, the fixed cells were centrifuged at 1000rpm for 5mins and stained with 5% propidium iodide (Med chemExpres, China). Finally, the cell cycle distribution was detected by Flow cytometry (CytoFLEX, USA).

### Cell apoptosis assay

Transfected cells were trypsinized and harvested. After washing twice with cold-PBS, cells were resuspended in 1× binding buffer at 1×10^6^ cells/mL. Next, 5ml Annexin V-PE and 10ml PI were added to the resuspended cells in accordance with the manufacturer's instructions. The cells were subsequently restored in the dark at 25 °C for 15 min. Finally, the cell apoptosis was determined by BD Accuri® C6 flow cytometer (BD biosciences, USA).

### Western blotting

We lysed transfected cells in RIPA buffer and extracted total protein. 10% SDS-PAGE was used to separate the total protein and we transferred the separated protein onto a PVDF membrane. Next, the protein was incubated in 5% skimmed milk (BBI, China) at 25 ℃ for 2 hours. Then, the membranes were incubated with primary antibodies overnight on ice, followed incubated with HRP-conjugated Affinipure Goat Anti-Mouse/Rabbit IgG for a total of 120 minutes at 37 °C. The protein bands on PVD membranes were determined by the chemiluminescence imaging system (BIO-RAD, USA) and the protein levels were measured by Image Lab software (BIO-RAD, USA).

### Statistical analysis

SPSS 23.0 and GraphPad Prism 8.0 software was employed to carry out statistical analysis and plot figures. The student's t-test was employed to compare the HCFC1 mRNA expression between HCC and adjacent normal tissues. The Chi-square test was used to analyze the association between HCFC1 protein level with clinicopathological parameters. Kaplan‐Meier analysis with the Log‐rank test was employed to compare survival rates. The Cox hazard regression model was employed to determine the predictive factors of survival and a recurrence value less than 0.05 means statistically significant.

## Results

### HCFC1 mRNA was upregulated and correlated with poor prognosis in HCC patients

**Figure 1:**
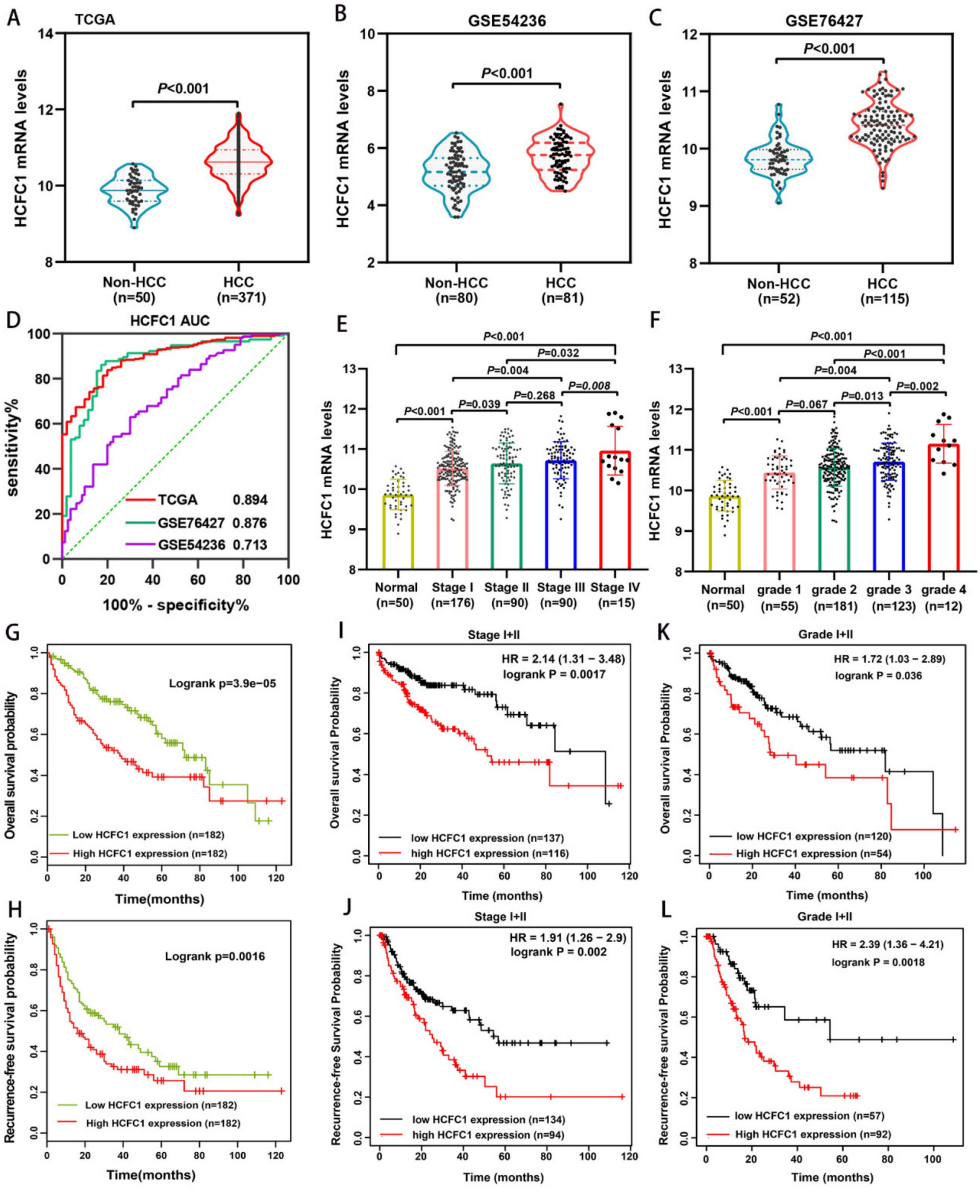
HCFC1 mRNA expression and its prognostic value in HCC. (A-C) HCFC1 mRNA was elevated in HCC compared with normal liver tissues in TCGA (A), GSE54236 (B), and GSE76427 (C) datasets. (D) The diagnostic value of HCFC1 was evaluated by the ROC curve in TCGA, GSE54236, and GSE76427 datasets. (E, F) HCFC1 mRNA was gradually increased with tumor stage (E) and grade (F) increasing. (G, H) High HCFC1 mRNA levels correlated with poor OS (G) and RFS (H). (I, J) High HCFC1 mRNA levels predicted poor OS (I) and RFS (J) in stage I/II patients. (K, L) High HCFC1 mRNA levels predicted poor OS (I) and RFS (J) in grade I/II patients.

We analyzed the HCFC1 mRNA level in TCGA and GEO (GSE54236 and GSE76427 dataset) databases and noticed that it significantly up-regulated in HCC compared with normal liver tissues (Figure 1A-C). In addition, the area under the curve (AUC) of receiver operating characteristics (ROC) analysis for three datasets was 0.894, 0.876, and 0.713, respectively, suggesting that HCFC1 mRNA has satisfactory prognostic significance for HCC (Figure 1D). Noteworthy, with tumor stage and grade increasing, HCFC1 mRNA was gradually increased (Figure 1E, F). Survival analyses were used to evaluate the prognostic significance of HCFC1 and demonstrated that high HCFC1 mRNA levels correlated with poor overall survival (OS) and recurrence-free survival (RFS) (Figure 1G, H). Furthermore, in HCC patients with early tumor stages (stage I+II) and grades (grade I+II), high HCFC1 mRNA expression still predicted an unsatisfactory prognosis (Figure 1I-L). We further investigated the clinical prognostic value of HCFC1 expression in patients with higher pathological stages and grades. The results showed that higher HCFC1 mRNA levels predicted poorer OS and RFS in patients with Stage III+IV and Grade III+IV (Supplementary Figure 1A, B).

### Higher HCFC1 protein correlated with poorer clinical outcomes and survival probability in a cohort of 150 HCC patients

**Figure 2:**
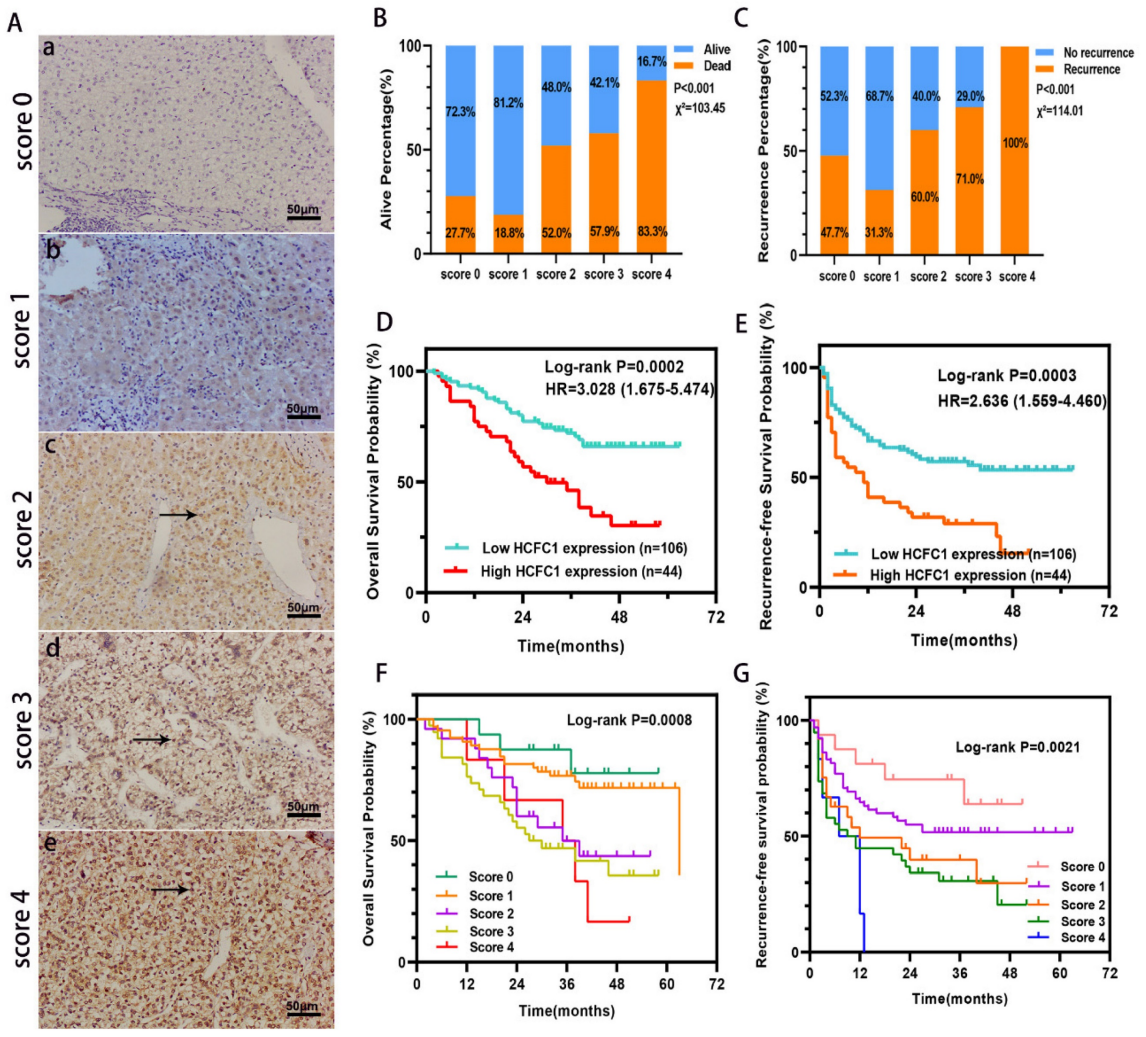
HCFC1 protein level and its prognostic value in a cohort of 150 HCC patients. (A) Representative images of HCFC1 IHC staining for scores of 0 (a), 1 (b), 2 (c), 3 (d), and 4 (e), respectively in HCC tissues. (Black arrows represented HCFC1 protein expression in the nucleus.) (B, C) The mortality (B) and recurrence rate (C) in patients with different HCFC1 IHC scores at the end of the follow-up period. (D, E) High HCFC1 protein expression predicts poor OS (D) and RFS (E). (F, G) OS (F) and RFS (G) curves of patients with different IHC staining scores.

HCFC1 protein expression was investigated in a cohort of 150 HCC patients and found that HCFC1 protein was significantly elevated in HCC compared with adjacent normal liver tissues. Representative images of different HCFC1 protein levels in HCC tissues were presented, and the HCFC1 protein was located predominantly in the nucleus (Figure 2A). At the end of the follow-up period, the mortality was 27.7%, 18.8%, 52%, 57.9%, and 83.3% for patients with a score of 0, 1, 2, 3, and 4, and the recurrence rate was 47.7%, 31.3%, 60%, 71%, and 100%, respectively (Figure 2B, C). We next stratified the 150 HCC patients into high and low -HCFC1 protein subgroups based on the IHC score (high HCFC1 subgroup: score of 3 and 4, low HCFC1 subgroup: score 0, 1, and 2). Correlation analysis suggested high HCFC1 protein was associated with higher tumor stage (P=0.049), larger tumor size (P=0.032), poor tumor differentiation (P=0.039), vascular invasion (P=0.005), higher recurrence rate (P=0.003) and mortality (P=0.001) (Table 2). Univariate Cox regression analysis elucidated that high HCFC1 protein was one of the risk factors for OS and RFS in HCC patients (Table 3). Moreover, multivariate Cox regression analysis confirmed that high HCFC1 protein was an independent risk factor for OS (aHR=1.868, 95%CI=1.064-3.279, P=0.030) and RFS (aHR=1.266, 95%CI=1.116-2.568, P=0.045) (Table 4). Furthermore, the K-M curve demonstrated that patients with high HCFC1 protein have poor OS and RFS compared with low HCFC1 expressed patients (Figure 2D, E). We also investigated the prognosis of patients with different IHC staining scores and found an increasing trend toward poor OS and RFS with the gradually increasing IHC score (Figure 2F, G).

### Higher HCFC1 protein also predicted poor prognosis in patients with early tumor stage, low tumor grade, and median/well differentiation

**Figure 3:**
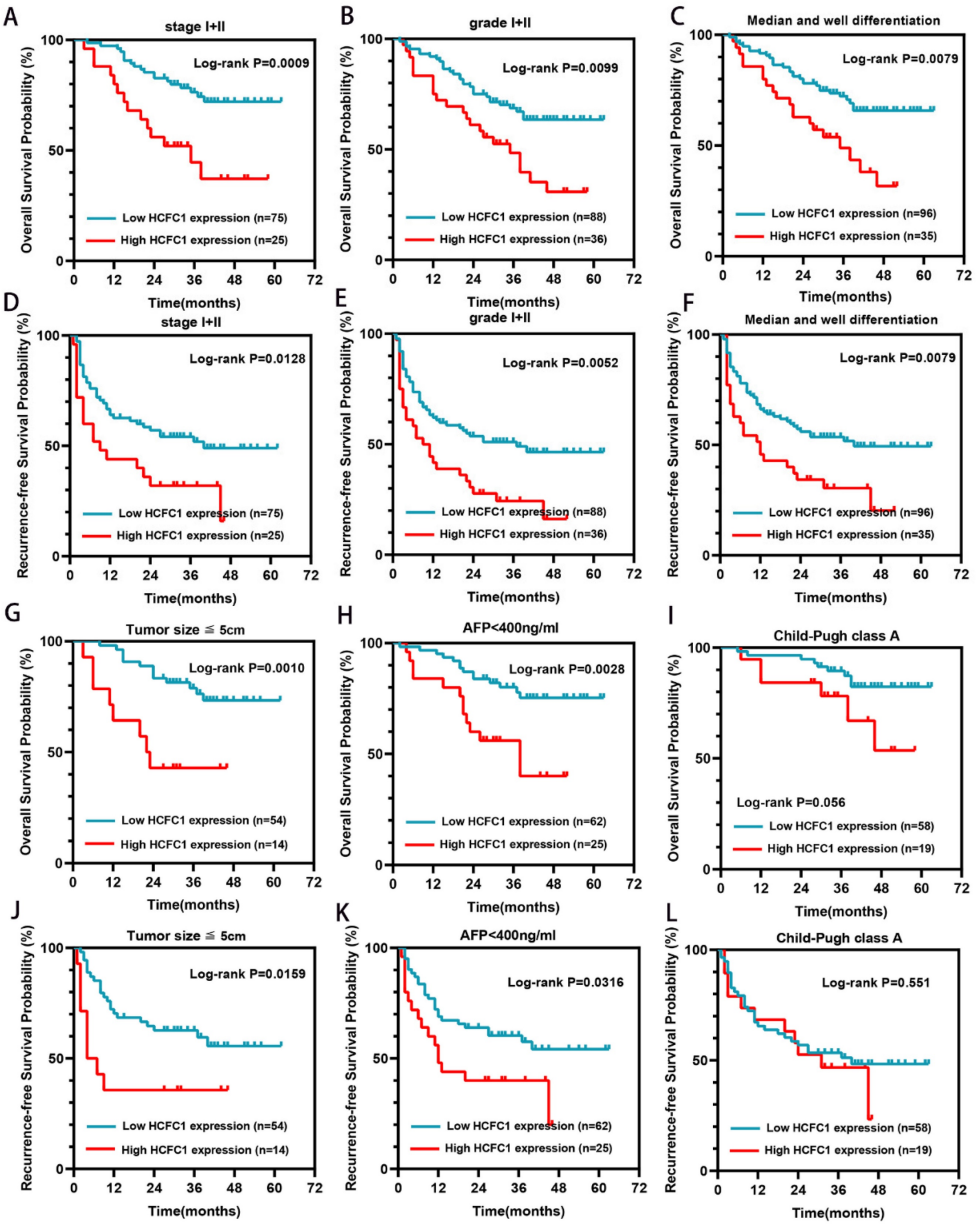
HCFC1 protein levels correlated to the prognosis of patients in early-stage subgroups. (A-C) High HCFC1 protein expression predicted shorter OS time for patients in stage I/II (A), grade I/II (B), and median/well differentiation (C) subgroups. (D-F) High HCFC1 protein expression predicted shorter RFS time for patients in stage I/II (D), grade I/II (E), and median/well differentiation (F) subgroups. (G-I) Overall survival probability of patients with different HCFC1protein levels in small tumor size (less than 5cm, G), low AFP level (less than 400ng/ml, H), and Child-Pugh class A (I) subgroups. (J-L) survival probability of patients with different HCFC1protein levels in small tumor size (less than 5cm, J), low AFP level (less than 400ng/ml, L), and Child-Pugh class A (L) subgroups.

Our further investigation elucidated that in the early tumor stage (I/II), low grade (I/II), median or well tumor differentiation, small tumor size (less than 5cm), and low alpha-fetoprotein (AFP) level subgroups (<400ng/ml), higher HCFC1 protein level patients both have shorter OS (Figure 3A-C, G-H) and RFS (Figure 3D-F, J-K) period than patients with lower HCFC1 protein expression (Figure 3). In Child-Pugh class A subgroups, both OS and RFS probabilities between high and low HCFC1 protein expression patients have no significant difference (Figure 3I, L).

### Higher HCFC1 mRNA expression was associated with higher TMB, MATH, MSI, and lower tumor purity levels in HCC

**Figure 4:**
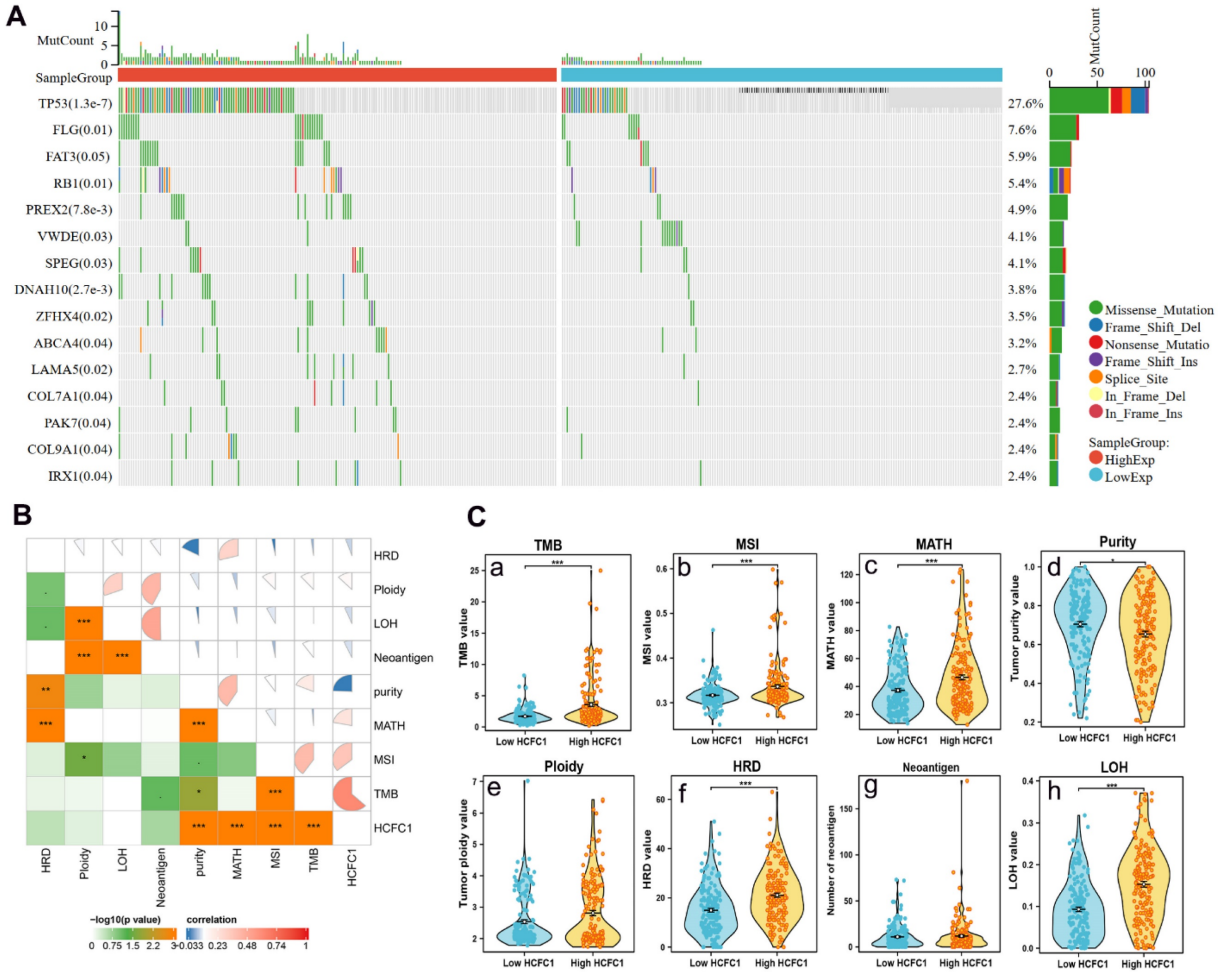
Differences in somatic mutations, TMB, and MSI Between HCFC1 high and low groups. (A) The mutation profiles exhibited the mutation difference of the top 15 genes in high and low HCFC1 expression groups. (B) High HCFC1 mRNA levels positively correlated with TMB, MATH, and MSI, whereas negatively correlated with tumor purity. (C) TMB (a), MSI (b), MATH (c), tumor purity (d), ploidy (e), HRD (f), Neoantigen (g), and LOH (h) values differ between high and low HCFC1 expression groups. *P < 0.05, **P < 0.01, ***P < 0.001.

Somatic mutation data of 369 HCC samples were downloaded from the TCGA database to investigate the associations between HCFC1 expression with mutation profiles. Mutation of the top 15 genes was detected in 178 (48.2%) HCC patients. Fisher's test was employed to compare the somatic mutation frequency difference between HCFC1 high and low groups. The waterfall diagram exhibited a significant mutation frequency difference of TP53, FLG, RB1, PREX2, ZFHX4, and LAMA5 (Figure 4A). Next, we investigate the associations between HCFC1 expression with TMB, MATH, MSI, tumor purity, tumor neoantigen, and homologous recombination deficiency (HRD). Results suggested that higher HCFC1 expression was associated with higher TMB, MATH, MSI, and lower tumor purity levels (Figure 4B). In addition, the HRD and loss of heterozygosity (LOH) of HCFC1 high-expression groups were significantly higher than HCFC1 low-expression groups (Figure 4C).

### HCFC1 mRNA expression correlated with immune cell infiltration and immune checkpoint inhibitor-related genes expression in HCC

**Figure 5:**
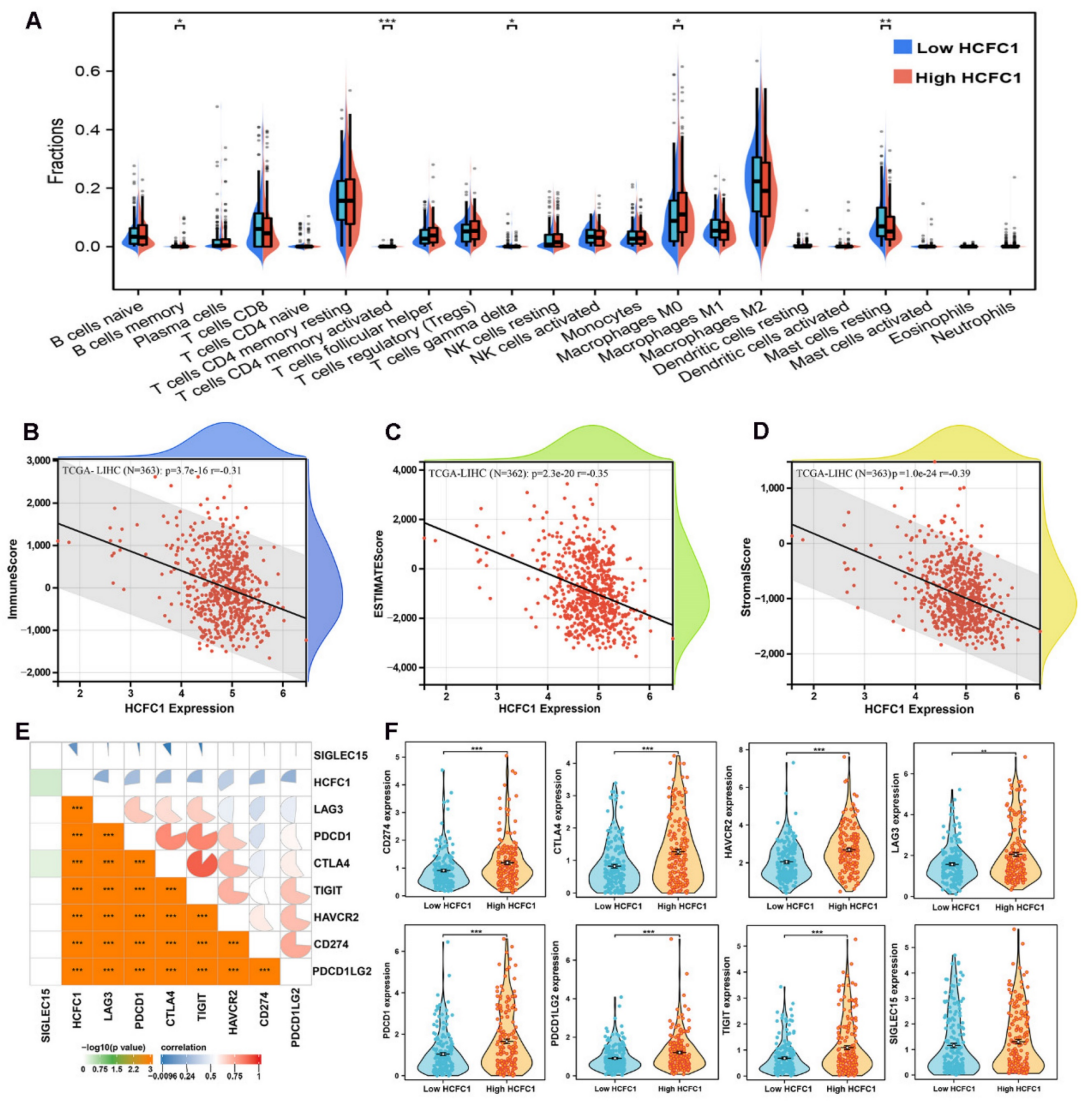
Association of HCFC1 and tumor immune characteristics in HCC. (A) The differential infiltrates abundance between HCFC1 high and low expression subgroups. (B-D) Correlations between HCFC1 expression with ImmuneScore (B), ESTIMATEScore (C), and Stromalscore (D) in HCC. (E) Correlations between HCFC1 expression and top 8 immune checkpoints inhibitor-related genes. (F) The expression difference of top 8 immune checkpoints inhibitor-related genes in HCFC1 high and low expression groups. *P < 0.05, **P < 0.01, ***P < 0.001.

It is generally accepted that the tumorigenesis and proliferation of HCC closely linked to the tumor immune microenvironment and immune cell infiltration 35, 36. Therefore, the CIBERSORT algorithm and single sample gene set enrichment analysis (ssGSEA) were employed to investigate the immune cell infiltration difference between HCFC1 high and low expression groups. The infiltration fractions of B cell memory, T cell CD4 memory, and macrophage M0 were remarkably higher, and the T cell gamma delta and mast cells resting were significantly lower in HCFC1 high expression groups (Figure 5A). Further, Pearson's correlation analysis revealed that HCFC1 expression significantly negatively correlated with the immune score, ESTIMATE score, and stromal score in HCC (Figure 5B-D). Next, we investigated Pearson's correlation between HCFC1 and immune checkpoint inhibitor-related genes expression and found that HCFC1 remarkably positively correlated with immune checkpoint inhibitor-related genes (Figure 5E). The expression of immune checkpoint inhibitor-related genes (CD274, CTLA4, HAVCR2, LAG3, PDCD1, PDCD1LG2, and TIGIT) was higher in HCFC1 high expression group (Figure 5F). Suggesting that higher HCFC1 expression patients may be resistant to immunotherapy and the inhibition of immune checkpoints may be novel treatment avenues for HCC patients.

### HCFC1 mRNA expression was significantly upregulated in HCC and immune cells at single-cell levels

**Figure 6:**
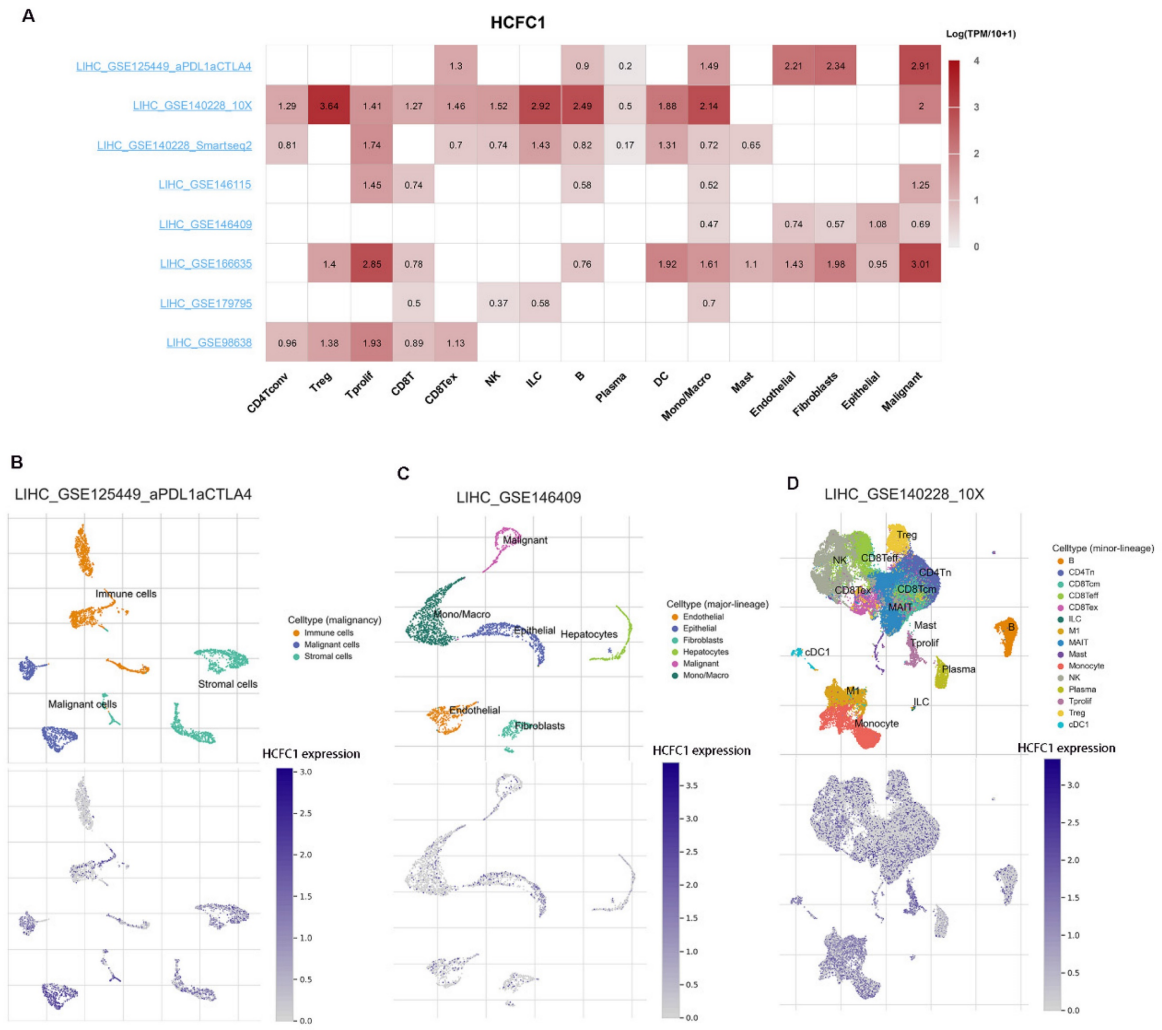
HCFC1 expression of single-cell RNA sequencing analysis. (A) In eight datasets, the average expression value of HCFC1 mRNA in different types of cells. (B) HCFC1 mRNA expression in malignant cells, immune cells, and stromal cells in the LIHC_GSE125449_aPDL1aCTLA4 dataset. (C) HCFC1 expression in normal hepatocytes, malignant cells, epithelial cells, and monocytes/macrophages in the LIHC_GSE146409 dataset. (D) The distribution of various immune cells and corresponding HCFC1 mRNA expression levels in the LIHC_GSE140228_10X dataset.

Eight datasets from the TISCH2 database were used to explore HCFC1 expressions at the single-cell levels (hepatocytes, malignant cells, stromal cells, and different immune cell types). The matrix heat map exhibited the average expression value of HCFC1 mRNA in different cell types (Figure 6A). In the LIHC_GSE125449_aPDL1aCTLA4 dataset, HCFC1 mRNA expression in malignant and immune cells was significantly higher than in stromal cells (Figure 6B). In addition, the normal hepatocytes exhibited a lower level of HCFC1 expression than malignant cells, epithelial cells, and monocytes/macrophages in the LIHC_GSE146409 dataset (Figure 6C). Figure 6D represented the distribution of various immune cells and corresponding HCFC1 mRNA expression levels in the LIHC_GSE140228_10X dataset, suggesting a higher level of HCFC1 expression in regulatory T cells, Monocytes/macrophages, and B cells, but a lower HCFC1 level in Mast cells. All results demonstrated that HCFC1 mRNA levels differ widely in different cell types and with a high level of malignant and immune cells, which may be the causes of the HCC immune microenvironment and tumor heterogeneity.

### HCFC1 Knockdown inhibited the proliferation and migration of HCC cells in vitro

**Figure 7:**
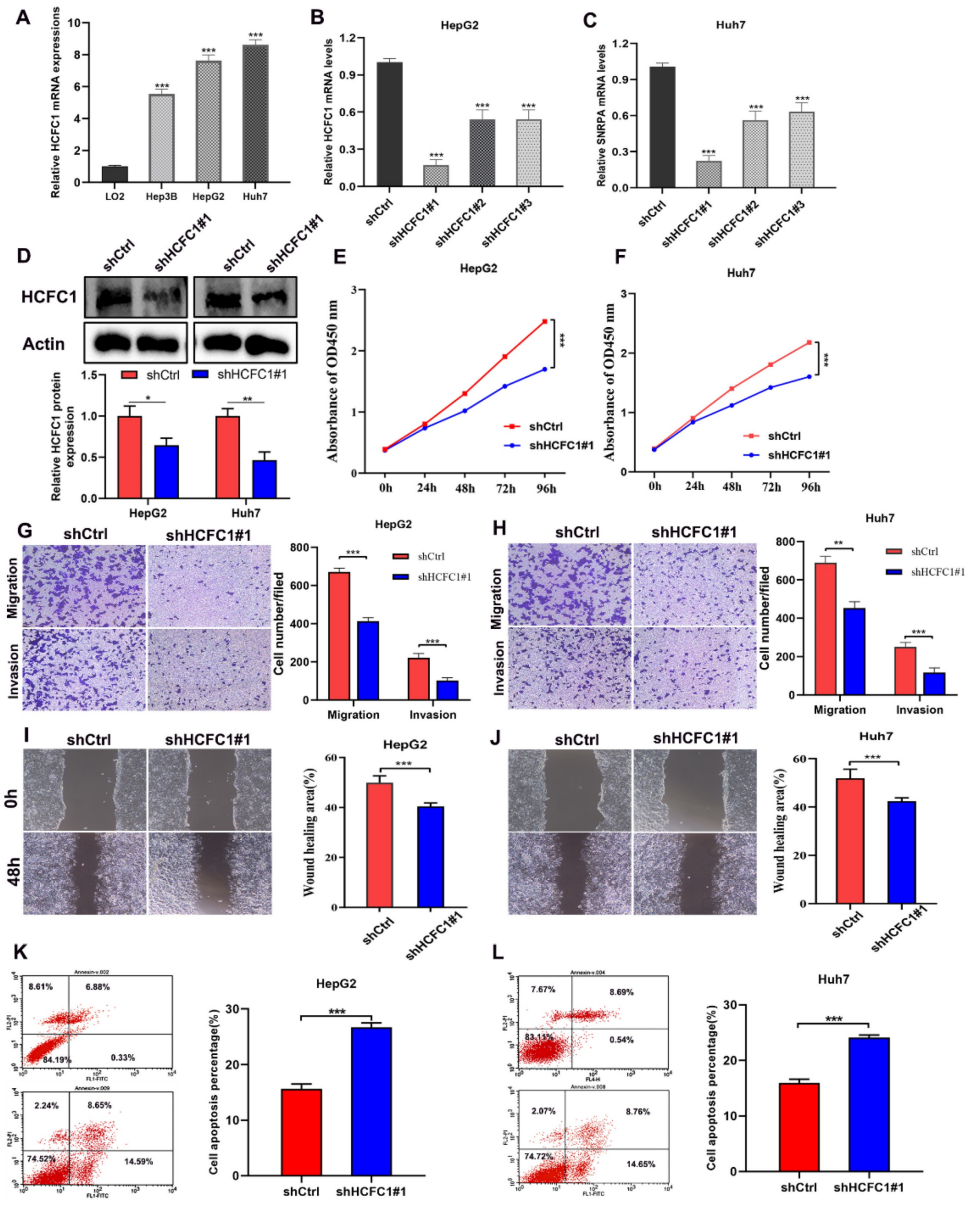
HCFC1 promoted the proliferation and migration of HCC cells. (A) HCFC1 expression was elevated in HCC cell lines compared with normal liver cells. (B, C) The HCFC1 mRNA level was decreased in HepG2 (B) and Huh7 (C) cell lines after transfected shHCFC1. (D) Western blot assay validated the inhibitory effects of shHCFC1#1 in HepG2 and Huh7 cells. (E, F) CCK-8 assays detected the knockdown of HCFC1 on HepG2 (E) and Huh7 (F) cell viability. (G, H) Representative images and quantified analysis of transwell assays in HepG2 (G) and Huh7 (H) cells of shCtrl and shHCFC1 groups. (I, J) HepG2 (I) and Huh7 (J) cells transfected with shHCFC1 exhibited higher migration capacity in wound healing a than with shCtrl cells. (K- L) HepG2 (K) and Huh7 (L) cells transfected with shHCFC1 had a higher apoptosis rate than cells transfected with shCtrl. **P < 0.01, ***P < 0.001. ns: no statistically significant.

We next clarify the effects of HCFC1 expression on tumor growth and biological behavior by conducting in vitro experiments. The qRT-PCR assay suggested higher HCFC1 mRNA levels in three HCC cell lines (Hep3B, HepG2, and Huh7) than in normal hepatocyte cell lines (LO2) (Figure 7A). HepG2 and Huh7 cells are found with higher HCFC1 mRNA expression than Hep3B cells in the qRT-PCR assay. We selected HepG2 and Huh7 cells for subsequent validation assays. Then, HCFC1 mRNA was knocked down by transfected lentiviral with targeted shHCFC1. The qRT-PCR determined the significant decrease of HCFC1 mRNA expression in two HCC cell lines (Figure 7B, C). shHCFC1#1 was selected in further experiments due to its highest knockout effects. Western blot assay further validated the inhibitory effects of shHCFC1#1 in HepG2 and Huh7 cells (Figure 7D). The CCK-8 assay revealed that the cell viability of shHCFC1#1 cells was significantly inhibited compared with shCtrl cells (Figure 7E, F). In addition, the transwell and wound healing assays indicated a significantly suppressed level of the migration and invasion capacity of HCFC1 knockdown cells compared with the shCtrl cells (Figure 7G- J). Moreover, flow cytometry assays illustrated that HepG2 and Huh7 cells transfected with shHCFC1 had a higher apoptosis rate than cells transfected with shCtrl (Figure 7K, L).

### Underlying biological functions and enrichment pathways of HCFC1

**Figure 8:**
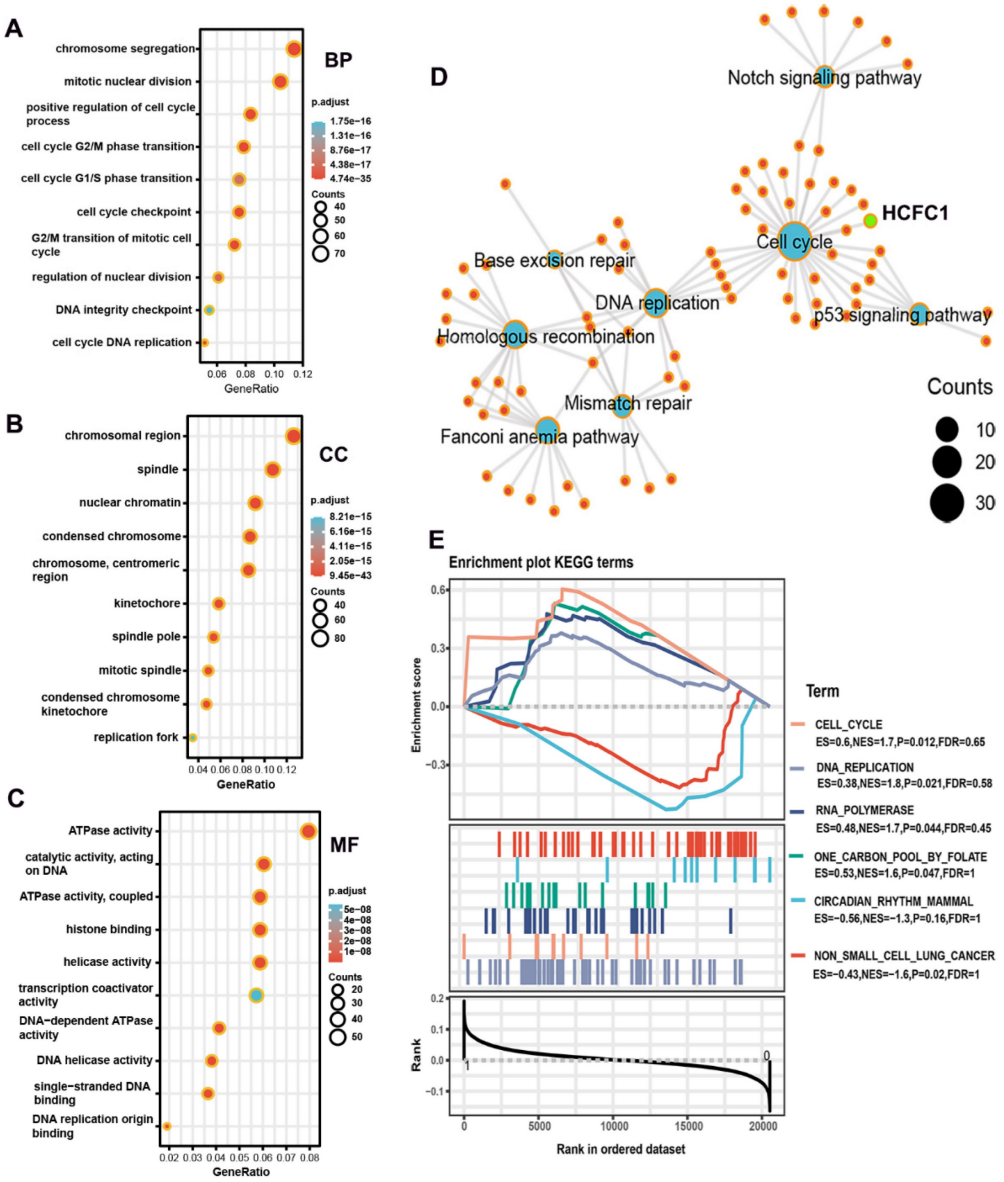
Underlying biological functions and enrichment pathways of HCFC1. (A-C) The biological process (A), cellular component (B), and molecular function (C) data in GO analysis. (D) The KEGG pathways of co-expressed genes with HCFC1. (E) GSEA results of significant signaling pathways enriched by high and low HCFC1 expression data sets enriched.

GO and KEGG analyses were performed on co-expressed genes of HCFC1 to identify the potential biological functions and signaling pathways. HCFC1 was associated with the cell cycle-related biological process, such as chromosome segregation, mitotic nuclear division, and positive regulation of the cell cycle process, etc (Figure 8A). In the cellular component terms, HCFC1 correlated to chromosomal region, spindle, nuclear chromatin, condensed chromosome, etc (Figure 8B). The molecular function of HCFC1 was mainly related to ATPase activity, catalytic activity, histone binding, helicase activity, etc (Figure 8C). In addition, the KEGG elucidated that HCFC1 was associated with the signaling of the Cell Cycle, P53 signaling pathway, DNA replication, Mismatch repair, etc (Figure 8D). Moreover, the GSEA analyses revealed that upregulated HCFC1 was shown to Cell Cycle, DNA replication, RNA polymerase, and One carbon pool by folate. Downregulation of HCFC1 correlated with Circadian rhythm mammal and non-small cell lung cancer (Figure 8E).

### HCFC1 knockdown induced cell cycle arrest in vitro

**Figure 9:**
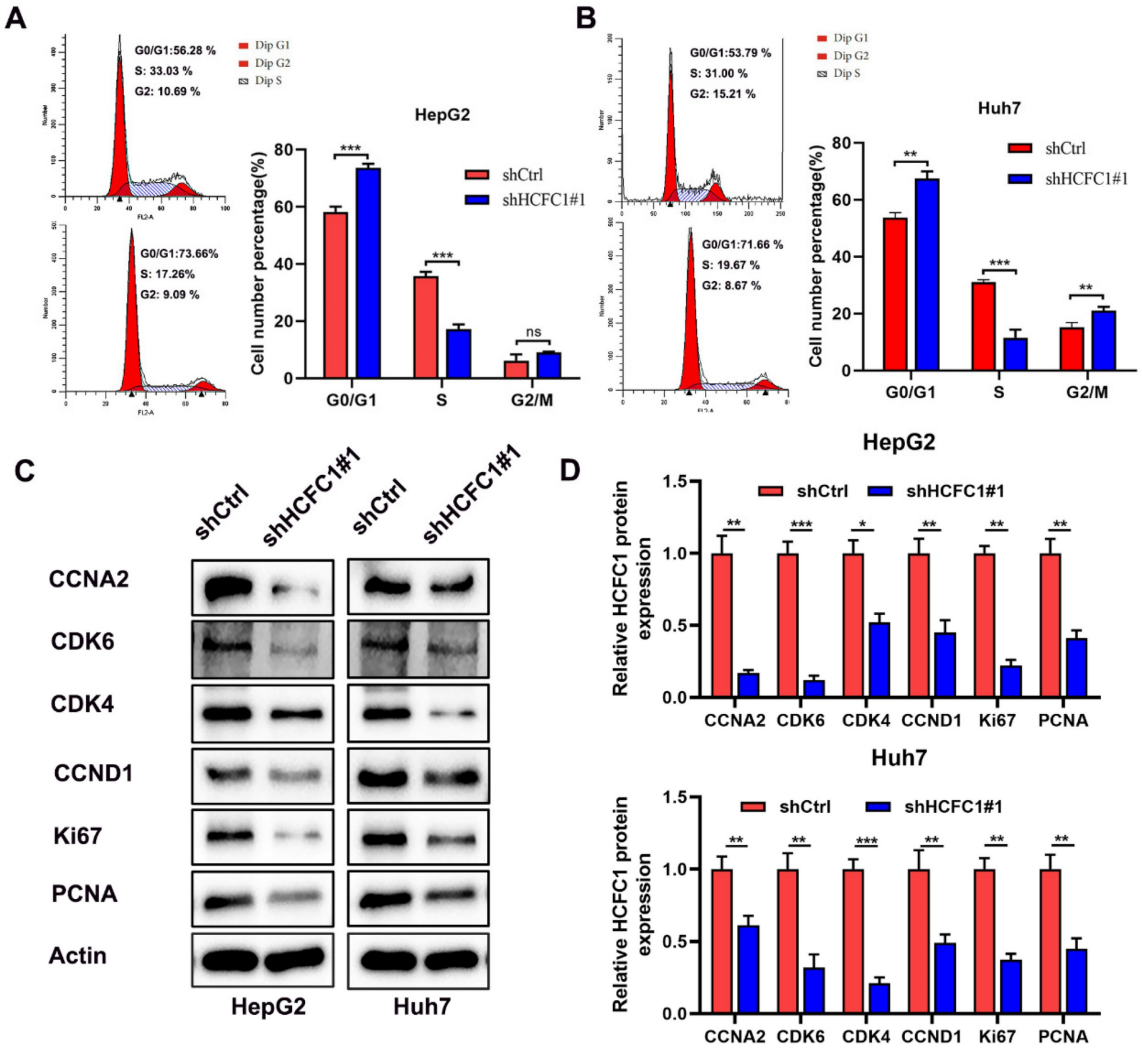
HCFC1 regulated the cell cycle of HCC cells. (A, B) The flow cytometry assays detect the cell cycle distribution of HepG2 (A) and Huh7 (B) cells. (C, D) Western blots images (C) and statistical analysis (D) of the protein expression of cell cycle-related markers in transfected HCC cells. *P < 0.05, **P < 0.01, ***P < 0.001.

The enrichment analyses suggested that HCFC1 was significantly linked to the pathway of cell cycle and chromosome segregation. We determined whether the cell cycle was affected by HCFC1 expression through vitro experiments. The flow cytometry assays demonstrated that HCFC1 knockdown arrested the HepG2 and Huh7 cells at G0/G1 phase and shortened the S phase (Figure 9A, B). We next investigated the protein expression of cell cycle-related markers in transfected HCC cells. Results demonstrated that protein levels of CCNA2, CDK6, CDK4, CCND1, Ki67, and PCNA significantly downregulated in shHCFC1 cells compared to the shCtrl cells (Figure 9C, D). Suggesting that HCFC1 regulated the progression of HCC through the cell cycle.

## Discussion

Our present study first put forward that HCFC1 mRNA and protein expression were elevated in HCC tissues and correlated to an unfavorite prognosis of HCC. Besides, HCFC1 high protein level was an independent risk factor for poor OS and RFS, especially for early-stage patients. Besides, elevated HCFC1 protein remarkably correlated to higher tumor stage, larger tumor size, poor tumor differentiation, vascular invasion, higher recurrence rate, and mortality. Upregulation of HCFC1 expression validated in qRT-PCR, Western blot, and IHC staining assays, suggesting that HCFC1 can function as oncogenes and promising prognostic biomarker.

HCFC1, a gene located on the X chromosome, activated early genes which regulated cell proliferation and metabolism by interacting with its VP16 transcription factor 37. The mutation of the HCFC1 gene domain in vitro disrupts binding to VP16, resulting in defective cell proliferation 38. Machida et al. reported that HCFC1 was deubiquitinated by the deubiquitinating enzyme BRCA1-associated protein 1 to regulate cell divisions and proliferation 39. In addition, HCFC1 has also been reported to interact with various proteins such as E2F and MALL to drive cell proliferation 18, 40. Likewise, HCFC1 exerts a pro-cancer effect in a variety of cancers by promoting cell division and tumor proliferation 41, 42. Itkonen et al. suggested that HCFC1 regulated cell cycle and proliferation in androgen-independent prostate cancer cells by interacting with MYC, an essential regulated mitotic protein 43. Our experiments in vitro validated that downregulation of HCFC1 expression inhibited cell viability, migration, and invasion capacity and promoted apoptosis, suggesting similar to other cancers, HCFC1 can serve as an oncogene and may be a novel target for treatment.

Bioinformatic analysis with KEGG demonstrated that co-expressed genes were mainly enriched in the cell cycle pathway. In addition, the GSEA analyses revealed that upregulated HCFC1 was shown to the Cell Cycle. The flow cytometry assays validated that HCFC1 knockdown arrested the HCC cells at G0/G1 phase and shortened the S phase, and the cell cycle-related markers were also downregulated. Here the assumption that HCFC1 promoted HCC proliferation by regulating the cell cycle pathway was reasonable.

Next, we investigated the associations between HCFC1 level and immune infiltration and found HCFC1 upregulation correlated to a higher proportion of B cell memory, T cell CD4 memory, and macrophage M0 but a lower proportion of T cell gamma delta and mast cells resting. Interestingly, the single-cell RNA sequencing analysis suggested that HCFC1 mRNA was elevated in these same immune cells. As we know, the progression of cancers was closely associated with the immune microenvironment and regulated by immune cell infiltration 44-46. The cell cycle was also linked to the anti-tumor immune response in a variety of types of cancers 47, 48. Deng et al. suggested that CDK4/6 enhanced T cell activation and augmented antitumor immunity in lymphoma 49. In addition, the combined application of CDK4/6 inhibitor and immune checkpoint inhibitors enhanced the anti-tumor efficacy of patients with small-cell lung cancer by promoting T cell activation 50. Our results point out that knockdown of HCFC1 expression significantly downregulated CDK4/6 expression in HCC cells, and low HCFC1 expression correlated with lower expression of immune checkpoint inhibitor-related genes and lower proportion of B cell memory, T cell CD4 memory. Therefore, we speculated that HCFC1 drives cell cycle regulation by affecting CDK4/6 and immune checkpoint genes. However, the specific mechanisms required further investigation.

## Conclusions

HCFC1 expression was upregulated in HCC tissues and predicted an undesirable prognosis, suggesting HCFC1 can be a promising diagnostic and prognostic biomarker for HCC patients. Aberrant HCFC1 expression was associated with mutation profiles and tumor immune microenvironment and immune cell infiltration. HCFC1 promoted the progression of HCC by regulating the cell cycle, and it could be a potential target for the design of individualized treatment strategies for HCC.

## Supplementary Material

Supplementary figure and table.

## Figures and Tables

**Table 1:** Primers sequences for qRT-PCR.

Name	Sequences
HCFC1	Forward: 5'-CGCCATATGGAGCTCCTC-3'
	Reverse: 5'-CCCTTCGATATGGTGATGG-3'
GAPDH	Forward: 5'-AAGGTGAAGGTCGGAGTCAAC-3'
	Reverse: 5'-GTTGAGGTCAATGAAGGGGTC-3'

**Table 2:** Correlation between HCFC1 protein expression and clinicopathologic features in 150 patients with hepatocellular carcinoma.

Characteristics			HCFC1 level		χ²	*P-Value
		N	high(n)	low(n)		
Age (year)	>55	98	32	66	1.503	0.220
	<=55	52	12	40		
Gender	Male	132	39	93	0.024	0.877
	Female	18	5	13		
Tumor size (cm)	>5cm	82	30	52	4.589	0.032
	<=5cm	68	14	54		
TNM stage	I/II	100	25	75	2.718	0.049
	III	50	19	31		
Serum AFP level	>400ng/ml	63	19	44	0.030	0.850
	<=400ng/ml	87	25	62		
Tumor location	Left	51	15	36	0.001	0.988
	Right	99	29	70		
Tumor differentiation	Poor	19	9	10	4.059	0.039
	Median	98	28	70		
	Well	33	7	26		
HBsAg	Positive	71	19	52	0.430	0.512
	Negative	79	25	54		
Edmonson grade	I/II	26	8	18	0.031	0.860
	III/IV	124	36	88		
Child-Pugh class	A	77	19	58	1.656	0.198
	B	73	25	48		
Vascular invasion	Yes	72	29	43	8.001	0.005
	No	78	15	63		
Tumor encapsulation	Yes	101	29	72	0.057	0.811
	No	49	15	34		
Recurrence	Yes	84	33	51	9.122	0.003
	No	66	11	55		
Survival status	Alive	89	17	72	11.054	0.001
	Dead	61	27	34		

TNM, tumor node metastasis; Aalpha-fetoproteintein. *P-Value<0.05 were considered statistically significant. A bold value is considered statistically significant.

**Table 3:** Univariate Cox Regression analysis of overall survival and recurrence-free survival in 150 patients with hepatocellular carcinoma.

Variables		Overall survival	*P-Value	Recurrence-free survival	*P-Value
		HR (95%CI)		HR (95%CI)	
Age (year)	>55 vs. <=55	0.995(0.589-1.681)	1.681	0.829(0.531-1.294)	0.409
Gender	Male vs. female	0.777(0.331-1.823)	0.563	1.354(0.734-2.496)	0.331
Tumor size (cm)	>5 vs. <=5	1.696(0.997-2.883)	0.051	1.742(1.117-2.717)	**0.014**
TNM stage	I/II vs. III	1.935(1.188-3.152)	**0.008**	1.299(0.831-2.031)	0.251
Serum AFP level	>400 vs <=400	1.838(1.105-3.057)	**0.019**	1.746(1.137-2.682)	**0.011**
Tumor location	Left vs. right	0.850(0.503-1.434)	0.502	1.306(0.818-2.085)	0.264
Tumor differentiation	Well vs. median/Poor	1.511(0.766-2.983)	0.234	1.811(1.018-3.211)	**0.043**
HBsAg	Positive vs. negative	1.288(0.776-2.137)	0.328	0.842(0.547-1.296)	0.435
Edmonson grade	I/II vs. III/IV	0.728(0.346-1.533)	0.404	0.756(0.410-1.393)	0.370
Child-Pugh class	A vs. B	5.103(2.827-9.213)	**<0.001**	1.566(1.016-2.413)	**0.042**
Vascular invasion	Yes vs. no	1.862(1.110-3.124)	**0.018**	2.308(1.486-3.585)	**<0.001**
Tumor encapsulation	Yes vs. no	0.887(0.522-1.510)	0.660	0.325(0.210-0.504)	**<0.001**
HCFC1 protein level	High vs. low	2.501(1.501-4.168)	**<0.001**	2.018(1.300-3.131)	**0.002**

HR, Hazard ratio; CI, confidential interval; TNM, tumor node metastasis; AFP, alpha-fetoprotein. *P-Value<0.05 were considered statistically significant. A bold value is considered statistically significant.

**Table 4:** Multivariate Cox Regression analysis of overall survival and recurrence-free survival in 150 patients with hepatocellular carcinoma.

Variables		Overall survival	*P-Value	Recurrence-free survival	*P-Value
		aHR (95%CI)		aHR (95%CI)	
Tumor size (cm)	>5 vs. <=5	0.894(0.448-1.787)	0.752	1.169(0.718-1.904)	0.531
TNM staging	I/II vs. III	0.879(0.459-1.683)	0.696		
Serum AFP level	>400 vs <=400	1.704(1.001-2.907)	**0.049**	1.582(1.013-2.471)	**0.044**
Tumor differentiation	Well vs. median/Poor			1.129(0.600-2.126)	0.707
Child-Pugh class	A vs. B	4.916(2.652-9.113)	**<0.001**	1.266(0.805-1.991)	0.308
Vascular invasion	Yes vs. no	1.526(0.867-2.685)	0.143	1.928(1.181-3.146)	**0.009**
Tumor encapsulation	Yes vs. no			0.330(0.209-0.522)	**<0.001**
HCFC1 protein level	High vs. low	1.868(1.064-3.279)	**0.030**	1.266(1.116-2.568)	**0.045**

aHR, adjusted Hazard ratio; CI, confidential interval; TNM, tumor node metastasis; AFP, alpha-fetoprotein. *P-Value<0.05 were considered statistically significant. A bold value is considered statistically significant.

## Abbreviations

HCFC1: Host cell factor 1
HCC: Hepatocellular carcinoma
TCGA: The Cancer Genome Atlas
TMB: Tumor mutational burden
MSI: Microsatellite instability
CCND1: Cyclin D1
CCNA2: Cyclin A2
CDK4: Cyclin-dependent kinase 4
CDK6: Cyclin-dependent kinase 6
GEO: Gene Expression Omnibus
IHC: Immunohistochemical
MATH: Mutant-allele tumor heterogeneity
HRD: Homologous recombination deficiency
TISCH2: Tumor Immune Single-cell Hub 2
TME: Tumor microenvironment
GO: Gene ontology
KEGG: The Kyoto Encyclopedia of Genes and Genomes
GSEA: Gene set enrichment analysis
shCtrl: shRNA-HCFC1 negative control
qRT-PCR: Quantitative polymerase chain reaction
GAPDH: Glyceraldehyde-3-phosphate dehydrogenase
CCK-8: Cell Counting Kit-8
OS: Overall survival
RFS: Recurrence-free survival
AFP: Alpha-fetoprotein
AUC: Area under the curve
ROC: Receiver operating characteristics
LOH: Loss of heterozygosity
ssGSEA: Single sample gene set enrichment analysis
