# Supplementary material for: Upregulation of HCFC1 expression promoted hepatocellular carcinoma progression through inhibiting cell cycle arrest and correlated with immune infiltration
Source: J Cancer. 2023 May 15;14(8):1381–97. doi: 10.7150/jca.84579 (PMC10240668; doi:10.7150/jca.84579)
Supplement: Supplementary file 1 — Supplementary figure and table. [file jcav14p1381s1.pdf]

Supplementary Figure 1. High HCFC1 expression predicted poor prognosis in HCC with higher stages (A) and grades (B).

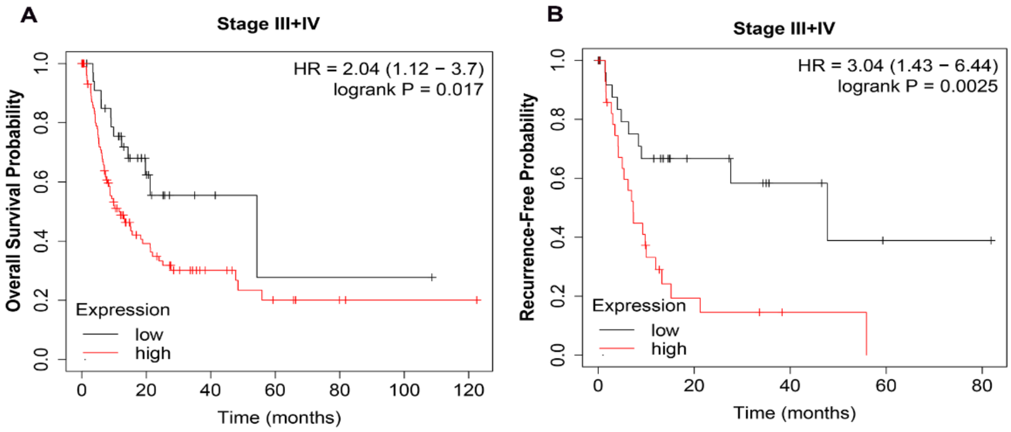

Supplementary Table 1. Primary antibodies for western blotting.

Protein	Concentration	Specificity	Company
HCFC1	1:1000	ab137618	Abcam
CDK6	1:1000	14052–1-AP	Proteintech
CDK4	1:1000	11026–1-AP	Proteintech
CCND1	1:2000	ab85247	Abcam
Ki67	1:1000	ab16667	Abcam
PCNA	1:1000	10205–2-AP	Proteintech
Cyclin A2	1:2000	66391–1-lg	Proteintech
